# Enhancing biodiversity: historical ecology and biogeography of the Santa Catalina Island ground squirrel, *Otospermophilus beecheyi nesioticus*

**DOI:** 10.1098/rsos.240726

**Published:** 2024-11-06

**Authors:** Torben C. Rick, Hugh D. Radde, Wendy G. Teeter, Emma A. Elliott Smith, Cindi M. Alvitre, Nihan D. Dagtas, Karimah O. Kennedy-Richardson, Julie L. King, Desireé R. Martinez, Stephanie Schnorr, Sabrina Shirazi, Jesús E. Maldonado, Courtney A. Hofman

**Affiliations:** ^1^ Department of Anthropology, National Museum of Natural History, Smithsonian Institution, Washington, DC 20013-7012, USA; ^2^ Repository for Archaeological and Ethnographic Collections, University of California Santa Barbara, Santa Barbara, CA 93106, USA; ^3^ Pimu Catalina Island Archaeology Project, Los Angeles, CA, USA; ^4^ Santa Ynez Band of Chumash Indians, Santa Ynez, CA, USA; ^5^ Department of Biology & Center for Stable Isotopes, University of New Mexico, Albuquerque, NM 87131, USA; ^6^ Ti’at Society/Traditional Council of Pimu, Avalon, CA, USA; ^7^ Department of Anthropology and Laboratories of Molecular Anthropology and Microbiome Research, University of Oklahoma, Norman, OK 73019, USA; ^8^ Department of Anthropology, University of California Riverside, Riverside, CA, USA; ^9^ Santa Catalina Island Conservancy, Avalon, CA 90704, USA; ^10^ Tribal Relations Office, California Polytechnic State University, Pomona, CA, USA; ^11^ Center for Conservation Genomics, Smithsonian’s National Zoo and Conservation Biology Institute, Washington, DC 20008, USA

**Keywords:** Islands, zooarchaeology, translocation, human-assisted dispersal, California

## Abstract

People have influenced Earth’s biodiversity for millennia, including numerous introductions of domestic and wild species to islands. Here, we explore the origins and ecology of the Santa Catalina Island ground squirrel (SCIGS; *Otospermophilus beecheyi nesioticus*), one of only five endemic terrestrial mammals found on California’s Santa Catalina Island. We synthesized all records of archaeological/palaeontological SCIGS, conducted radiocarbon dating and stable isotope analysis of the potentially earliest SCIGS remains and performed genetic analysis of modern SCIGS. Squirrels were not identified in island palaeontological deposits, but at least 12 island archaeological sites contain SCIGS bones, including some that are butchered or burned. All directly dated SCIGS bones are Late Holocene in age and younger than approximately 1290 cal BP. The first mitochondrial genome for modern *Otospermophilus beecheyi* and 15 modern SCIGS mitogenomes document at least one introduction of squirrels. Stable isotope data indicate variable SCIGS diets and potential subsidies from marine environments to terrestrial plants consumed by some individuals. We cannot rule out a natural overwater dispersal, but the earliest SCIGS remains post-date the earliest evidence for people by several millennia and, along with other lines of evidence, support a human-assisted translocation of squirrels during the Late Holocene. These data illustrate the important role of Indigenous people in shaping and enhancing island biodiversity and ecology around the world.

## 1. Introduction

People’s activities have been intertwined with the world’s biodiversity for millennia, including the introduction of a variety of wild and domestic species to new areas [1–5]. While several key examples of island translocations exist and span some 20 000 years, biological introductions by hunter–gatherers are generally less well documented than those of agricultural groups [3,6,7]. This leaves a gap in our understanding of the relationships between biodiversity, human activities and environmental change.

Here, we focus on the origins and ecology of the Santa Catalina Island ground squirrel (SCIGS, *Otospermophilus beecheyi nesioticus*), one of only five endemic terrestrial mammals on Santa Catalina Island (SCAI, *Pimungna* in the Tongva language), California, USA [8]. Isolated from the adjacent mainland throughout the Pleistocene, Santa Catalina is one of California’s eight Channel Islands, all of which contain unique biodiversity and long records of Indigenous settlement [9–11]. Today, SCIGS is abundant, found across the entire island, serves a variety of ecological functions (e.g. seed/nut dispersal and as prey for rattlesnakes and other animals) and is viewed by some island residents as pests [12,13]. Despite the widespread occurrence of SCIGS and speculation that they were introduced by Tongva (Gabrielino) peoples [8,14,15], when and how squirrels first arrived on SCAI remain open questions. Current archaeological data suggest a colonization after approximately 5900 years ago and perhaps much later, but to our knowledge, no squirrel bones have been directly accelerator mass spectrometry (AMS) radiocarbon dated and no genetic analyses of SCIGS have been performed to date [15,16].

**Figure 1.**
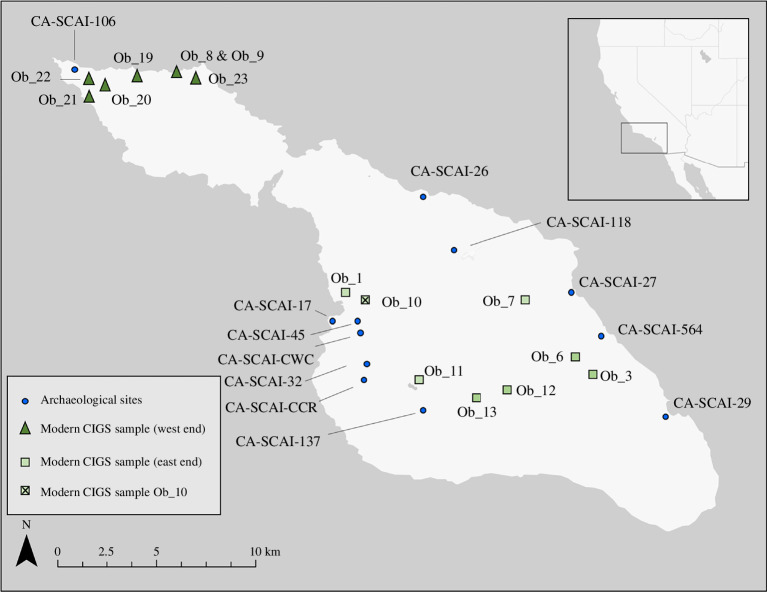
Location of archaeological sites (blue circles) with SCIGS bones and modern genetic samples (green triangles and squares) on Santa Catalina Island. All sites contain direct radiocarbon dates on SCIGS bones except CA-SCAI-29, CA-SCAI-32, CA-SCAI-564 and CA-SCAI-CWC. Inset of western United States with box around area containing Santa Catalina Island.

In this article, we synthesize the occurrence of SCIGS remains in archaeological sites, document evidence for burning or processing of archaeological SCIGS bones, present the first direct AMS radiocarbon dates and stable isotope data from archaeological SCIGS and discuss the first genetic analysis of modern SCIGS (figure 1). Our work sought a multi-proxy approach [3,5,17] using genetic (ancient and modern), stable isotope and direct radiocarbon analyses to understand the timing and implications of SCIGS colonization of the island. Because of unsuccessful ancient DNA analysis, our study pivoted to focus on genetic analyses of modern SCIGS samples. We use these multi-proxy data to explore the origins and antiquity of SCIGS, genetic relationships among island and mainland squirrels*,* the dietary niche of SCIGS through time and the relationships between Tongva people and insular squirrels and ecosystems. When placed in regional and global context, these data provide key insights for understanding the role of people in shaping and enhancing biodiversity in the past, present and future.

## 2. Context and background

Santa Catalina is part of the unceded territories of the Tongva peoples. Radiocarbon dating from across the island demonstrates that people lived on SCAI for at least 6000 years [10,11]. Continued radiocarbon dating on Santa Catalina will probably produce radiocarbon dates older than 6000 years ago, a proposition supported by the identification of chipped stone crescents that generally date to before 8000 years ago on SCAI, and the well documented presence of people on nearby San Clemente Island for approximately 9000 years and the northern Channel Islands for at least 13 000 years [10,18]. Today, SCAI is the only one of the Channel Islands with permanent settlement, with a community of residents based mainly at Avalon, which is the island’s primary town a. As Teeter *et al*. [11, p. 160] noted, ‘Santa Catalina Island has been, and continues to be, an important hub for the intermingling of people and the exchange of ideas and objects.’ In the past, this was facilitated by the *ti’at* or plank canoe that was used to transport people and materials between the islands and mainland, while a network of trails guided the movement of people, ideas and objects across the island landscape [11].

Along with San Nicolas, Santa Barbara and San Clemente islands, Santa Catalina is one of the southern Channel Islands. Located 32 km from the mainland coast, Santa Catalina is 194 km^2^ in area making it the third largest of the Channel Islands. The island has a relatively rugged terrain, with its highest peak at 670 m, and a Mediterranean climate, with an average daytime high of approximately 21°C and rainfall averaging about 30 cm yr^−1^ [8,11].

Like all the Channel Islands, Santa Catalina was never connected to the mainland during the Pleistocene. The water gap separating the island from the mainland resulted in a somewhat distinct flora and fauna relative to the adjacent mainland, including a much smaller number of terrestrial mammals [8,14]. For instance, only five endemic mammals (excluding bats) are found on SCAI: SCAI fox (*Urocyon littoralis catalinae*), SCAI harvest mouse (*Reithrodontomys megalotis catalinae*), SCAI deer mouse (*Peromyscus maniculatus catalinae*), SCAI shrew (*Sorex ornatus willetti*) and SCIGS. Yet Santa Catalina has the highest endemic mammalian diversity of all the Channel Islands. Several researchers have argued that some of these mammals and other organisms were introduced to Santa Catalina and/or other Channel Islands by people during the Holocene, including the island fox, harvest and deer mouse, SCIGS, island lizards and perhaps spotted skunks [14,15,19–28].

**Figure 2.**
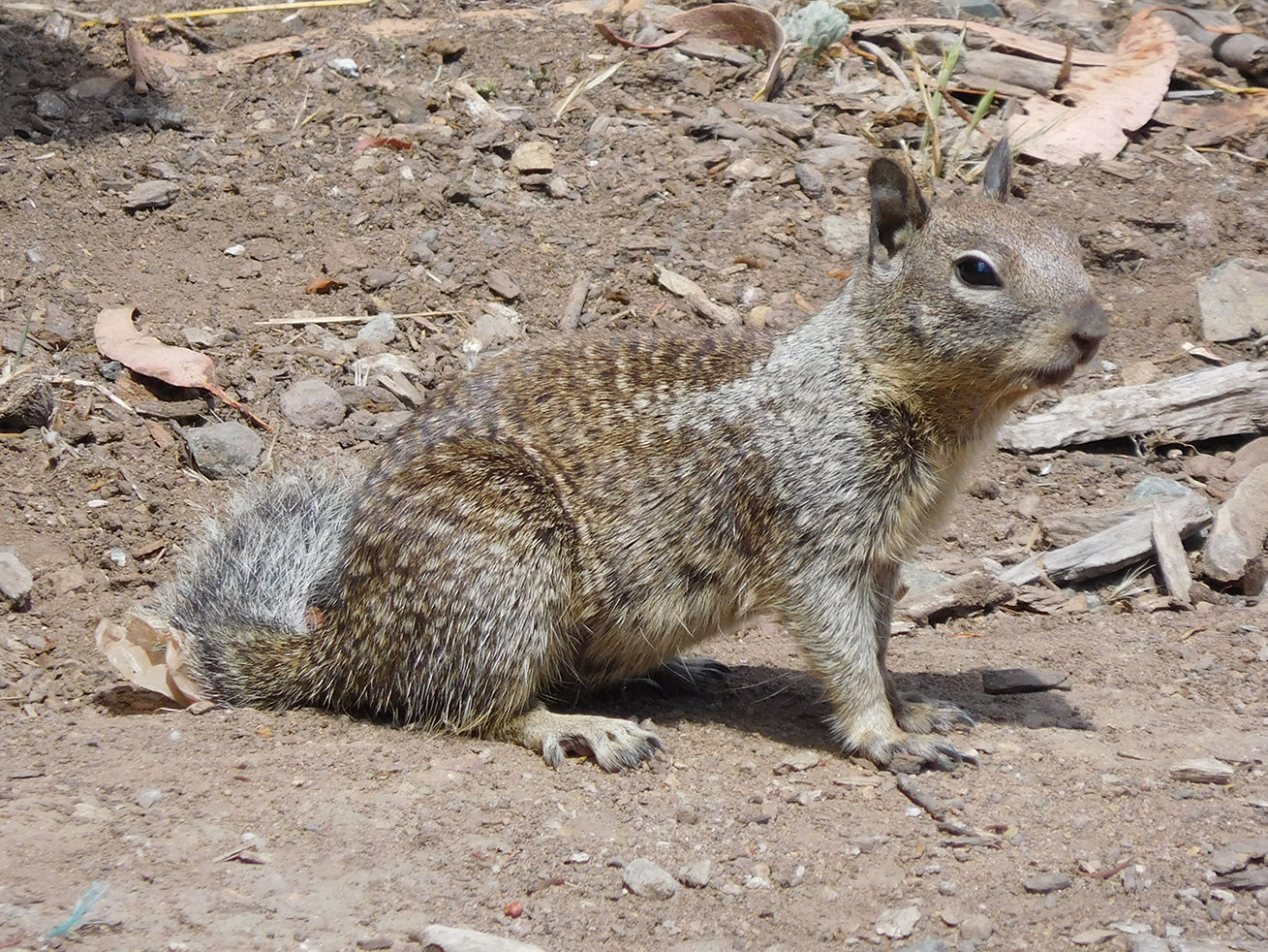
Santa Catalina Island ground squirrel (photo by Julie King).

SCIGS is thought to be closely related to the California ground squirrel (*Otospermophilus beecheyi*) found on the adjacent mainland [29]. Most of what is reported in the literature about the biology of SCIGS comes from early research during the first half of the twentieth century or slightly later [30]. These studies determined that mainland and island *O. beecheyi* are very similar, with primary differences being a slightly larger size for SCIGS and an apparently darker colour (figure 2 [31, pp. 263–264], [32, pp. 49–51], [33, pp. 160–161], [13, p. 254]). The SCIGS eats a wide variety of foods, including insects, fruits and grasses, are found in diverse habitats across the island (from the seacoast to mountain peaks) and fill a similar niche as mainland ground squirrels [13,32]. No genetic studies have yet been conducted on SCIGS, and consequently, the focus of determining their history and colonization patterns has been based on archaeological evidence and possible palaeontological occurrences on Santa Catalina.

Prior to our study, researchers relied on a literature review, documenting the presence of SCIGS bones in at least six archaeological sites (CA-SCAI-17, CA-SCAI-45, CA-SCAI-118, CA-SCAI-137, Camp Cactus Road and Cottonwood Creek) [15, p. 53]. These archaeological SCIGS remains have been discovered in Late Holocene (4200 cal BP to present) and Middle Holocene (8200 to 4200 cal BP) archaeological sites based on associated radiocarbon dates or the presence of time sensitive artefacts [15,34–40]. Evidence for butchering and burning of archaeological squirrel bones suggests considerable human interaction [25,41]. However, to our knowledge, there are no palaeontological records of SCIGS, with the exception of squirrel bones found in modern bald eagle nests on the island [42]. SCIGS does not occur on any of the other Channel Islands, but Braje *et al*. [43, p. 34] reported the introduction of the California ground squirrel to San Nicolas Island in 1994 and that population has since disappeared.

## 3. Material and methods

### 3.1. Santa Catalina Island ground squirrel occurrence data and sample selection

We compiled all previous occurrence records of ancient SCIGS in the literature with H.D.R. and W.G.T. also searching for specimens in museums/repositories and adding a few additional sites to the previous known occurrence data, including CA-SCAI-26, CA-SCAI-27 and CA-SCAI-106 (table 1). Several sites with SCIGS, such as CA-SCAI-17, CA-SCAI-26, CA-SCAI-27, CA-SCAI-29, CA-SCAI-106 and CA-SCAI-564, were major Tongva villages [11, p. 158]. We also noted any reported evidence for bone modification, especially cut marks or evidence of burning (e.g. charred/blackened bone). Because only some of the bones in a given site were burned, this burning is inferred to be from human processing rather than a natural fire. From this review and analysis, we selected eight SCIGS bones from across all of these sites, particularly those that could be of the greatest antiquity (table 2). The preservation status of these bones was evaluated before they were prepared for AMS and stable isotope analysis.

**Table 1.** Archaeological sites containing squirrel bones on Santa Catalina Island, including number of identified specimens (NISP) and evidence for cultural modification (cut marks and burning). (NR, not reported.)

site	age^a^	NISP	NISP %^b^	elements with cut marks (%)	elements with burning (%)	reference	
CA-SCAI-17	5990–1185 cal BP	17	0.4	NR	NR	[35]	
CA-SCAI-26	430–130 cal BP	363	6.9	0	24 (7)	[36]	
CA-SCAI-27	900–465 cal BP	138	7.4	2 (1)	12 (9)	[25]	
CA-SCAI-29	2345–550 cal BP	717	19.3	0	304 (43)	[34]	
CA-SCAI-32	6165–480 cal BP	447	34.1	0	24 (5)	[37]	
CA-SCAI-45	1450-1050 BP	25	13.8	0	0	[40]	
CA-SCAI-106	1365–540 cal BP	19	0.3	0	0	[39]	
CA-SCAI-118	450–150 BP	12	37.5	NR	NR	[40]	
CA-SCAI-137	670–380 cal BP	126	19.3	present	1 (1)	[41]	
CA-SCAI-564	1515–130 cal BP	47	9.8	0	3 (6)	[38]	
CA-SCAI-Camp Cactus Road (CCR)	665–365 cal BP	154	86.0	NR	NR	[41]	
CA-SCAI-Cottonwood Creek (CWC)	3680–3270 cal BP	1	0.8	NR	NR	[44]	

^a^ Chronology follows Radde *et al.* [10] .

^b^ Percentage for CA-SCAI-17 does not include birds or fishes and for CA-SCAI-32 they do not include fish bone. Similarly for Camp Cactus Road the percentage is only based on mammals and is probably very overinflated.

**Table 2.** Radiocarbon and stable isotope data for Santa Catalina Island ground squirrels.

site and age^a^	provenience	element	laboratory number	C : N_atomic_	δ^13^C	δ^15^N	^14^C age	calibrated age (BP, 95%)^b^
CA-SCAI-17 (5990–1185 cal BP)	pit 25, 10–20 cm (acc 417)	mandible	OxA−29781	3.3	−19.0	8.4	214 ± 24	305–0
CA-SCAI-26 (430–130 cal BP)	acc 18, unit 0S/3W, 50–60 cm	vertebra (subadult)	UCIAMS−239781	3.2	−19.9	15.3	385 ± 15	500–330
CA-SCAI-27 (900–465 cal BP)	acc 563, cat 3397, unit 8, 30–40 cm	mandible	UCIAMS−239782	3.2	−20.7	4.7	675 ± 15	670–565
CA-SCAI-45 (1450-1050 BP)	pit 1, 30–40 cm	innominate	OxA−29783	3.2	−18.7	14.8	395 ± 23	510–325
CA-SCAI-106 (1365–540 cal BP)	acc 851, cat 4268, unit E1, 10–20 cm	humerus	UCIAMS−239780	3.2	−20.5	4.9	1290 ± 15	1285–1175
CA-SCAI-118 (450-150 BP)	pit D5, 12–18 cm	femur (subadult)	OxA−29784	3.3	−22.8	6.0	119 ± 23	275–10
CA-SCAI-137 (670–380 cal BP)	unit 7, L4, 30–40 cm	tibia	OxA−29780	3.3	−19.4	3.7	609 ± 25	650–545
CA-SCAI-Camp Cactus Road (CCR) (665–365 cal BP)	unit 3, 30–40 cm	mandible	OxA−29782	3.3	−19.4	2.7	656 ± 24	670–555

^a^ Calibrated age ranges are for site deposits obtained from Radde *et al.* [10]. For CA-SCAI-26 an end date of 130 cal BP was used.

^b^ Calibrated with OxCal 4.4 using the IntCal20 database.

### 3.2. Stable isotope analysis and radiocarbon dating

Five of the archaeological SCIGS samples were sent to the Oxford Radiocarbon Accelerator Unit at the University of Oxford, and three additional samples were sent to the Keck Carbon Cycle AMS Facility at the University of California Irvine for AMS radiocarbon and stable isotope analysis. Each laboratory processed and pretreated the samples using ultrafiltration techniques, and both facilities are well versed in preparing and radiocarbon dating archaeological bones. The Oxford and Keck laboratories removed separate subsamples for δ^13^C and δ^15^N analysis. For these isotopic analyses, all samples and reference materials were calibrated against internationally accepted standards: Vienna-Pee Dee Belemnite (V-PDB; δ^13^C) and atmospheric N_2_ (δ^15^N). Isotopic results are presented as delta values (δ) in parts per thousand (‰), where δ^13^C or δ^15^N = 1000*[(*R*_samp_/*R*_std_) - 1]. *R*_samp_ and *R*_std_ are the ^13^C :^12^C or ^15^N :^14^N ratios of sample and standard, respectively [45].

Because measured radiocarbon ages differ from calendar ages, all radiocarbon dates were calibrated using OxCal 4.4 [46] and treating all of the SCIGS as terrestrial samples by applying the Intcal20 calibration curve [47]. In a meta-analysis of radiocarbon dates from SCAI, Radde *et al.* [25] applied an approximate end date of 130 cal BP (AD 1820), an age roughly corresponding with Indigenous depopulation of the island, to any dates with calibrated ranges extending beyond that time period. This is probably true for the calibrated ages presented in this study. However, because squirrel bones could also be intrusive (e.g. younger animals that burrowed into or had their bones mixed by a disturbance into older deposits) in the archaeological deposits in which they were recovered, we have not imposed an end date here.

### 3.3. Genetic analyses

Between 2013 and 2016, staff from the SCAI Conservancy collected 23 tissue samples from SCIGS, primarily from roadkill or individuals accidentally captured in traps intended for island foxes (electronic supplementary material, tables S1 and S2). From these, complete mitogenomes were successfully sequenced for 15 individuals (see the electronic supplementary material for detailed methods and Genbank Accession numbers). To our knowledge, this is the first time that mitogenomes have been published for any *O. beecheyi* subspecies.

After rigorous quality filtering, our novel mitogenomes, each comprising 16 471 base pairs (bp), were aligned to conduct a phylogeographical analysis. However, owing to the challenge of lacking comprehensive whole mitogenome data from California mainland populations for direct comparisons, we strategically extracted partial alignments of 795 bp of the mitochondrial cytochrome *b* gene (cyt *b*). This allowed for the incorporation of comparative mitochondrial data from previous studies by Phuong *et al.* [48] and Álvarez-Castañeda & Cortés-Calva [49], which included cyt *b* haplotypes from a broad range of mainland localities. By integrating these cyt *b* haplotypes, we were able to construct a more robust comparative framework, enriching our analysis of SCIGS in relation to their mainland counterparts.

To visualize the genetic relationships and potential evolutionary pathways among the sampled SCIGS, we generated a median joining network in PopArt v. 1.7 with the mitogenomes. Additionally, for the phylogeographical analysis, our study used the cyt *b* alignment (approx. 795 bp, *n* = 154). The selection of an appropriate substitution model (TPM2u+F+G4) determined by the Bayesian information criterion score was conducted using IQTree v. 2.2.2.3. Maximum likelihood phylogenies were then estimated with IQTree using 10 000 ultrafast bootstrap (UFboot) replicates with SH-aLRT.

## 4. Results

### 4.1. Archaeological occurrences and bone modification

We identified 12 archaeological sites with SCIGS, including a total of 2066 squirrel bones (table 1). While squirrel bones generally represent a minor component of the total vertebrate faunal collections at these sites, contributing about 15% or less of the total vertebrate or mammalian assemblages, they constitute a larger fraction at a few sites (e.g. CA-SCAI-32 and CA-SCAI-118). However, it is important to note that interpreting the precise abundance of squirrel bones can be challenging owing to the varied ways different researchers present data, with some only presenting total SCIGS count and not including the skeletal element identifications and counts that are needed to determine minimum number of individuals. A considerable number of the squirrel bones found at some sites are thought to be intrusive, with researchers speculating the bones come from a more recent or modern individual (table 1). Not all researchers reported evidence of bone modification, yet evidence exists of cut marks on at least two squirrel bones, with indication from another site that cut marks are also present there. However, 368 bones show signs of having been burned, including one assemblage (CA-SCAI-29) where 43% of all SCIGS bones exhibit signs of being burned.

### 4.2. Chronology and stable isotope ecology

**Figure 3.**
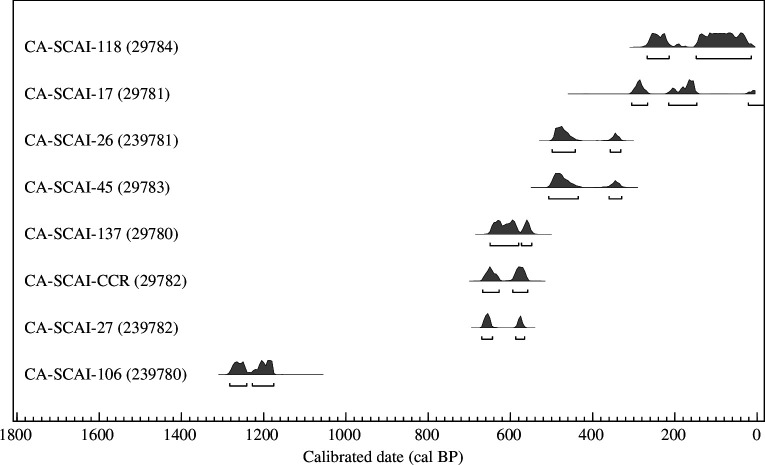
Radiocarbon age distributions for SCIGS specimens reported in table 2. Each date listed by radiocarbon sample number and archaeological site. These are 95% confidence intervals for each date and the unmodelled calibrated age using the IntCal20 terrestrial calibration curve.

Despite the potential for some SCIGS samples to be as old as approximately 5900 years, samples from all eight archaeological sites returned direct dates younger than 1290 cal BP (table 2; figure 3). The oldest squirrel bone in our sample is from CA-SCAI-106 at 1285–1175 cal BP. Three specimens from CA-SCAI-27, CA-SCAI-137 and CA-SCAI-CCR (Camp Cactus Road) were dated between about 670 and 545 cal BP. Bones from CA-SCAI-26 and CA-SCAI-45 were about 510 and 325 cal BP, with the samples from CA-SCAI-17 and CA-SCAI-118 dated to approximately 310 cal BP to potentially modern times. When compared to the associated chronologies for these sites, all but one of the dates falls within the established site chronologies [10]. The date from CA-SCAI-17 is a few centuries younger than other dates for this site, suggesting it might be intrusive in the deposits or related to an undocumented site component. A similar argument was made for Torqua Cave (CA-SCAI-32), where Porcasi [37] documented evidence for bioturbation and site disturbance, supported in part by the presence of squirrel bone in the assemblage. We were unable to date bone from Torqua Cave, but the majority of squirrel remains identified at this site come from the upper strata 0–120 centimetres below the surface (cmbs) and nearly absent from the oldest strata (120–230 cmbs), reinforcing the hypothesis for a Late Holocene arrival on island.

**Figure 4.**
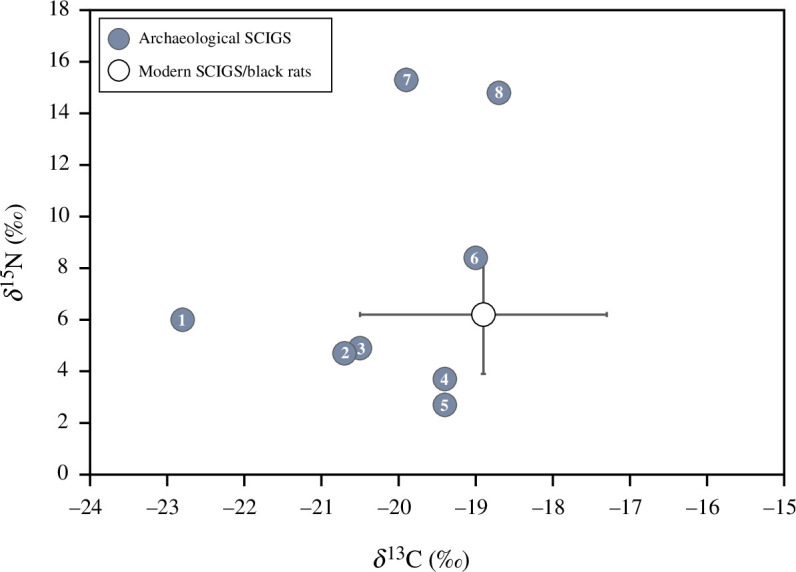
Bulk bone collagen δ^13^C and δ^15^N values of archaeological and modern Santa Catalina Island ground squirrels (SCIGS; *Otospermophilus beecheyi nesioticus*). Filled circles are isotopic results for archaeological SCIGS specimens from eight Santa Catalina Island archaeological sites: (1) CA-SCAI-118, (2) CA-SCAI-27, (3) CA-SCAI-106, (4) CA-SCAI-137, (5) CA-SCAI-CCR (camp cactus road), (6) CA-SCAI-17, (7) CA-SCAI-26 and (8) CA-SCAI-45. Open circle represents mean δ^13^C and δ ^15^N values of modern ground squirrels (*n* = 9) and black rats (*n* = 4) from Newsome *et al.* [42]; error bars show standard deviation around the mean. These data come from bulk tissue analysis of bone collagen extracted from remains found in bald eagle nests on Santa Catalina Island. Following Dombrosky [51], we have corrected the modern SCIGS/black rat δ^13^C data for temporal shifts in δ^13^C values of atmospheric CO_2_ (i.e. the Suess effect) by adding 1.8 ‰.

Atomic [C] : [N] values of all eight archaeological SCIGS fall within the accepted range of well preserved bone collagen, from 2.8 to 3.6 [50]. Isotopic values of SCIGS bulk bone collagen range from −22.8 ‰ to −18.7 ‰ (δ^13^C) and+2.7 ‰ to +15.3 ‰ (δ^15^N). The measured isotopic values of five SCIGS specimens are within the range of previously reported isotopic values of modern Santa Catalina rodents, including SCIGS [42], after accounting for temporal changes in δ^13^C values (figure 4).

### 4.3. Mitogenomes and phylogeny

The use of mitochondrial DNA (mtDNA) has proven to be extremely powerful for inferring genealogical and evolutionary relationships among and within populations and species [52,53]. Analysis of mtDNA offers a particularly rich source of markers for the study of closely related taxa because of the very low rate of recombination, maternal inheritance, simple genetic structure, reduced effective population size (Ne) and relatively rapid rates of evolution [52,54,55]. In addition, since mtDNA frequently evolves faster than nuclear DNA and is inherited maternally without recombination, it makes it stable over generations allowing researchers to trace maternal lineages and study migrations over time periods [7].

**Figure 5.**
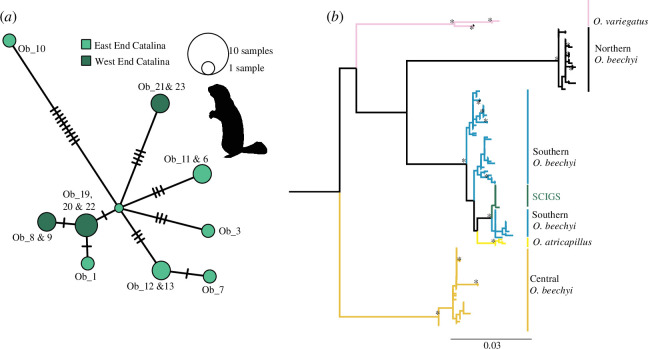
Genetic analysis of Santa Catalina Island ground squirrels (SCIGS). (*a*) Median joining network analysis of mitochondrial genomes from present-day SCIGS. The star-shaped pattern supports a single introduction. However, the divergent haplotype, Ob_10, may represent an additional introduction. (*b*) Maximum likelihood phylogeny with IQTree of partial cytochrome *b* gene (approx. 795 bp) with comparative data from [48] and [49]. Nodes with UFboot values 95% and above and SH-aLRT values above 80% are labelled with stars to indicate strong support. Following [48], the tree was rooted with *Callospermophilus lateralis* (NC_031210 not shown). SCIGS fall within the southern *O. beechyi* group and are closely related to *O. beechyi* sampled in southern California and Baja.

We attempted to recover mtDNA from all eight of the ancient samples that we directly radiocarbon dated but were unsuccessful in obtaining such material (electronic supplementary material, table S3). Mitochondrial genomes were successfully recovered from 15 modern SCIGS, representing samples from both the western and eastern ends of the island (electronic supplementary material, table S2; figure 5). From these samples, we recovered 10 closely related mitogenome haplotypes. Our analysis also revealed a total of 27 segregating sites across the entire 16 471 bp of the mitogenomes, with 12 sites being parsimony informative indicative of low genetic diversity in this population. Notably, one sample (Ob_10) stood out owing to its significant divergence, with more than 11 mutations between the next closest haplotype (figure 5*a*).

Further analysis, which included extracting and comparing a partial cyt *b* gene (795 bp) from the SCIGS mitogenomes to those from mainland localities, provided additional insights and showed that the Santa Catalina samples collapse into just two haplotypes. Both of these haplotypes form a monophyletic clade that is closely related with strong support (UFboot values 95% and above and SH-aLRT values above 80%) to the southern clade of *O. beecheyi* (figure 5*b*). While SCGIS group most closely with squirrels sampled in the San Bernardino National Forest and Baja California, Mexico (electronic supplementary material, table S4), we are hesitant to identify a possible source population for SCIGS given the limitations of this partial cyt *b* gene analysis ([22]; see the electronic supplementary material). In fact, Ob_10 collapses into the majority haplotype in this phylogenetic analysis, and Ob_1 is the only SCIGS sequence that is variable across this 795 bp fragment.

## 5. Discussion

### 5.1. Santa Catalina Island ground squirrel biogeography, translocation and genetics

Zooarchaeological analyses and direct radiocarbon dating of SCIGS bones demonstrate that ground squirrels colonized SCAI by at least 1290 cal BP. Recognizing that the squirrel bones dated here do not necessarily represent the earliest living squirrels on Santa Catalina, the available archaeological and genetic evidence suggests that ground squirrels probably appeared during the Late Holocene (4200 cal BP to present), perhaps during the latter half of this period. The relative morphological similarities between mainland and island ground squirrels noted by naturalists and biologists [13,32] are consistent with a relatively recent arrival. While SCIGS is slightly larger than their mainland counterparts, research on Channel Islands mice shows that rapid size changes can occur in as little as a century [56]. This rapid size change documented in Channel Island mice fits well within the Late Holocene arrival date for squirrels to SCAI, supporting relatively rapid size increases among SCIGS. For other California squirrel populations (not including SCIGS), body size variation across the Pleistocene is thought to correlate with precipitation rather than temperature or other variables, providing an important area for future research on SCIGS [57]. In the Gulf of Alaska, Indigenous people may have introduced Arctic ground squirrels (*Urocitellus parryii*) to Chirikof Island, demonstrating that ground squirrel translocations possibly occurred in the deep past elsewhere in the Americas [7].

The results of the genetic analyses provide further insights into the evolutionary history, colonization patterns and genetic diversity of SCIGS. The star-shaped median joining network analysis (figure 5*a*) shows genetic homogeneity and suggests a relatively recent common ancestry for the island’s ground squirrel population. Phylogeographical analysis (figure 5*b*) reveals that the samples from SCAI form a distinct, monophyletic clade consisting of two cyt *b* haplotypes. This clade exhibits a closer, well-supported genetic relationship with one of the southern clades of *O. beecheyi* found on the mainland. This finding strongly suggests a closer phylogenetic relationship between the SCIGS population and ground squirrel populations from southern California and Baja. Moreover, the clustering of Santa Catalina samples into a single monophyletic clade supports the hypothesis of a single introduction or colonization event for SCIGS on SCAI and that it probably originated from a source population in southern California. The distinctiveness of the mitogenome from sample Ob_10, collected in 2014 in a popular campground near Little Harbor, is noteworthy, especially as another sample, Ob_1, was collected from the same campground in 2013 and is part of the star-shaped cluster (figure 5*a*). This suggests that the colonization history may be more complex, with a possible second introduction, but this merits further investigation to more fully understand the genetic landscape of SCIGS. Additional island samples and comparative mitogenome data from mainland samples are necessary to test the hypothesis of a second introduction as there may be unsampled lineages in either location.

Establishing the precise timing of the genetic divergence between island and mainland populations remains challenging without additional mitochondrial genomes from mainland localities. Downsampling the mitogenome data to a short fragment of the cyt *b* to match the available comparative data from the mainland resulted in a loss of variation, complicating the determination of divergence times. However, given the relatively low level of genetic divergence observed in SCIGS mitogenomes across the western and eastern ends of the island, coupled with the fast generation time of rodents, our results support a Late Holocene introduction consistent with the archaeological data. The genetic and radiocarbon data which point to an arrival of squirrels several millennia after people, as well as burned and butchered squirrel bones in archaeological sites, and well-documented maritime trade and interaction between SCAI, the mainland, and other Channel Islands all support a human-assisted translocation of squirrels to Santa Catalina by Tongva peoples. Although we cannot rule out a natural over-water dispersal of squirrels during the Late Holocene, a chance introduction of a pregnant squirrel or breeding pair and subsequent establishment of a population several millennia after human arrival is unlikely given the current genetic and archaeological evidence.

Why would people introduce squirrels to SCAI? While it remains possible that a squirrel introduction could have been by stowaways hiding in a boat as has long been hypothesized for mice and rats around the world [58], this seems unlikely for SCIGS. Radde (2023) has recently reported a variety of zooarchaeological analyses for Santa Catalina, determining that SCIGS bones increase in relative abundance across three sites from 1365 to 130 cal BP. Burning and butchering of SCIGS bones [41] have long been reported, indicating that one reason for an introduction was for food (table 1). Given the large amount of marine foods available to Tongva peoples and present in the archaeological record [25,35,39], using squirrels as a food source was not likely because of protein scarcity on the island, but could have been something that people desired to eat. Among southern California Native American tribes, small mammals, such as squirrels, were known to have been roasted in earth ovens, cooked directly over fire and pulverized in mortars for stews [59–61]. In addition to the presence of burned/butchered SCIGS bones, rodent protein residues were identified on the surface of a groundstone bowl from CA-SCAI-29 and offer additional insight to the ways that squirrels may have been included in Tongva diets [34].

Squirrel pelts, along with island fox and sea otter pelts, would have been important resources for people as well. In the Gulf of Alaska, people made coats out of squirrel pelts [7] and that could have been a practice on SCAI [40]. Finally, since SCIGS is ubiquitous across mainland California, they would have been a familiar component of the landscape to people and a likely animal suited for translocation.

Tongva people had complex and far-reaching interaction spheres both via boats and terrestrial trails and corridors on the mainland and islands [11,25]. On Santa Catalina and the other Channel Islands, this was facilitated by the *ti’at* (plank canoe) that transported people and goods from outside of the island, including obsidian and deer bone from the mainland, soapstone from Santa Catalina to the mainland and other islands and more [11,62]. Squirrels could have been a component of this exchange and transport, as well as the translocation of other species, including the harvest mouse [21] and the island fox that appears to have been introduced by people probably from the northern Channel Islands by about 6000 years ago [22,23,26]. The timing of the first appearance of the *ti’at* has been debated with some speculating they first appeared around 1500 years ago [63], a date that corresponds well with the earliest squirrel dates. Evidence for a variety of sophisticated maritime activities on Santa Catalina like tuna harvest predate 1500 cal BP by a few millennia, suggesting a potentially earlier use of the *ti’at* [25]. Regardless of the exact timing of the use of the plank canoe, people used reed boats, dugouts or other boats well prior to this time—all of which could have been a mode of introducing squirrels and other animals to SCAI.

### 5.2. Santa Catalina Island ground squirrel diet and ecology

The stable isotope data presented here provide further insight into the ecology of Late Holocene SCIGS, demonstrating that squirrels had diverse diets and foraged across a range of microhabitats on Santa Catalina (figure 4). The δ^13^C and δ^15^N values of the SCIGS specimens from CA-SCAI-17, CA-SCAI-27, CA-SCAI-106, CA-SCAI-137 and CA-SCAI-CCR (Camp Cactus Road) indicate a diet of predominantly terrestrial C_3_ vegetation, with potential contribution from CAM vegetation. This inference is supported by historical accounts noting heavy consumption of seeds and corms, particularly those of *Dipterostemon capitatus* (blue dicks) by SCIGS, as well as extensive burrowing among cactus fields [32]. The elevated *δ*^15^N value (+8.4 ‰) of the specimen from CA-SCAI-17, relative to other SCIGS sampled here, may reflect a lactation signal given that this individual is a juvenile [22,64].

Three obvious outliers are present in our isotopic SCIGS dataset. The specimen from CA-SCAI-118 has lower δ^13^C values than other archaeological and modern samples by approximately 2 ‰ (figure 4). This could indicate less consumption of CAM plants by this individual relative to other SCIGS. However, given the potentially modern age of this specimen (table 2), it is also possible that the low δ^13^C value is an artefact of temporal shifts in the δ^13^C value of atmospheric CO_2_ over the past 200 years—i.e. the ‘Suess effect’ [51]. Two additional SCIGS specimens, from sites CA-SCAI-26 and CA-SCAI-45, have anomalously high δ^15^N values of+15.3 ‰ and+14.8 ‰, respectively, nearly 10 ‰ higher than the average of other SCIGS specimens.

One explanation for these isotopic values is scavenging by SCIGS on marine foods in or around villages. However, the mean bone collagen δ^13^C values of modern marine ichthyo- and avifauna from Santa Catalina are substantially higher (−17.0 ‰ to −14.3 ‰ [42]) than those measured here for SCIGS. This discrepancy makes it unlikely that these ground squirrels were foraging directly on marine resources. Instead, we argue the SCIGS from CA-SCAI-26 and CA-SCAI-45 was probably foraging on terrestrial vegetation fertilized by marine nitrogen. On Santa Barbara Island, similar patterns of high (‘marine’) δ^15^N values and low (‘terrestrial’) δ^13^C values have been documented for endemic *Peromyscus*, owing to consumption of vegetation fertilized by seabird guano [65]. Although the northern Channel Islands generally have higher numbers of breeding seabirds, there are several species that historically or currently breed on Santa Catalina [42,66], making this explanation plausible.

## 6. Conclusion

The probable introduction of squirrels to SCAI is just one aspect of a variety of activities undertaken by Tongva people that fit into the broader creation of cultural landscapes, including the legacies of trails, hunting, fishing and gathering activities, rock art and the creation of villages and other settlements [11]. SCAI is a land shaped by Tongva people with those legacies still present today and often unrecognized by the casual visitor or resident. Among their contributions to the island’s ecosystems, the Tongva people actively enhanced biodiversity through the probable introduction of several mammals including some of its most iconic non-domesticated inhabitants: squirrels, foxes and mice [21,22]. While some researchers and managers indicate that the close connections between foxes (and potentially other Channel Island fauna) and Tongva and Chumash peoples should challenge their uniqueness or need for conservation [67], we disagree. The intimate connections between Indigenous people and island fauna make these mammals more significant and worthy of protection. It also obviates the profound role of Tongva people in shaping the ecosystems and biodiversity of their homelands, including active enhancement of some of its most important species (e.g. foxes and squirrels). While conservationists, resource managers, the public and Indigenous communities work to restore Santa Catalina and other Channel Island ecosystems, it is imperative that the relationships between Indigenous peoples and island biodiversity inform future decision-making processes. In this sense, the probable translocation of squirrels during the Late Holocene helps understand their relationships with people in the past and present, which is key to SCIGS management and integration into broader island ecological restoration efforts [68]. Moreover, there is an important social justice dimension to these endeavours. While efforts continue to restore endemic fauna and eradicate invasive species that were introduced during the ranching and modern periods and create a more ‘natural landscape’, equal attention should be devoted to restoring the connections between Tongva peoples and SCAI. By acknowledging, honouring and incorporating Indigenous perspectives, histories and contributions to the island’s ecology, we can implement a more inclusive and sustainable approach to conservation and restoration efforts [69].

Beyond SCAI, people have had a strong role in shaping Earth’s ecosystems for thousands of years, including Indigenous stewardship that enhanced ecosystems through the use of fire, construction of clam gardens and a wide range of other activities [2]. People introduced a variety of domesticated and wild species to new areas, sometimes resulting in environmental degradation and extinction [3,5,6,58]. However, people also enhanced biodiversity as part of larger environmental engineering and landscape stewardship and as one component of creating a cultural landscape [1–3]. Globally, scores of introduced species have been documented through zooarchaeological research and investigated using genetic analyses, stable isotope analyses, radiocarbon dating and other analytical tools [1,3,5]. These studies have shown the fallacy of arbitrary divisions between people and nature and the blurred lines between wild and domesticated species [17]. Islands have played a particularly important role in these studies as the bounded landscapes can often be used to help discern the timing of species arrivals and introductions [3,5,58,70]. While agriculturalists are the best-known examples of introductions, including Neolithic dispersals in the Mediterranean, Norse and earlier occupations in the North Atlantic, and Polynesian introductions throughout the Pacific, hunter–gatherers began translocating mammals to islands some 20 000 years ago in island southeast Asia and Melanesia [58,71].

The term hunter–gatherer is a misnomer in many ways since people had diverse and complex land use and management strategies that transcend simple divisions between agriculture and foraging [72]. However, the translocation and introduction of mammals and other species by predominately hunter–gatherer groups remain poorly understood, particularly in North America. The California Channel Islands were a centre of this type of activity, with evidence for the introduction of both wild carnivores and rodents, domesticated dogs and herpetofauna, with other endemic fauna arriving via natural dispersal [14–16,19,20,22,26,27]. Similarly in the Caribbean, several translocations associated with horticulturalists have been documented [5,6,73,74]. These are probably just the tip of the iceberg in terms of the introductions and broader landscape influence that Indigenous people had throughout the Americas. Future research should focus on investigating other translocations, especially reptiles/amphibians, plants and other species that can help broaden our understanding of the intersection between people and North American biodiversity and ecology. As the work on SCIGS demonstrates, Indigenous people created cultural landscapes spanning centuries and millennia, and future management must prioritize the restoration and conservation of biological and cultural landscapes as the two are deeply intertwined.

## Data Availability

All data for this article are available in the tables and text or within the electronic supplementary material and tables S1, S2, S3. SCIGS mitogenome sequences are available on NCBI Genbank. All unused portions of the archaeological bones sampled for radiocarbon dating and stable isotope and genetic analysis were returned to the Catalina Island Museum for curation. Supplementary material is available online [75].
